# Order from entropy: big data from FAIR data cohorts in the digital age of plant breeding

**DOI:** 10.1007/s00122-025-05040-5

**Published:** 2025-09-24

**Authors:** Abhishek Gogna, Daniel Arend, Sebastian Beier, Ehsan Eyshi Rezaei, Tobias Würschum, Yusheng Zhao, Jianting Chu, Jochen C. Reif

**Affiliations:** 1 Leibniz Institute of Plant Genetics and Crop Plant Research, Corrensstraße 3, 06466 Gatersleben, Germany; 2 Institute of Bio- and Geosciences (IBG-4: Bioinformatics), Bioeconomy Science Center (BioSC), CEPLAS, Forschungszentrum Jülich GmbH, Wilhelm-Johnen-Straße, 52428 Jülich, Germany; 3 Leibniz Centre for Agricultural Landscape Research, Eberswalder Straße 84, 15374 Müncheberg, Germany; 4 Institute of Plant Breeding, Seed Science and Population Genetics, University of Hohenheim, 70599 Stuttgart, Germany

## Abstract

Lack of interoperable datasets in plant breeding research creates an innovation bottleneck, requiring additional effort to integrate diverse datasets—if access is possible at all. Handling of plant breeding data and metadata must, therefore, change toward adopting practices that promote openness, collaboration, standardization, ethical data sharing, sustainability, and transparency of provenance and methodology. FAIR Digital Objects, which build on research data infrastructures and FAIR principles, offer a path to address this interoperability crisis, yet their adoption remains in its infancy. In the present work, we identify data sharing practices in the plant breeding domain as Data Cohorts and establish their connection to FAIR Digital Objects. We further link these cohorts to broader research infrastructures and propose a Data Trustee model for federated data sharing. With this we aim to push the boundaries of data management, often viewed as the last step in plant breeding research, to an ongoing process to enable future innovations in the field.

## Introduction

Genomic prediction has helped shape breeding programs toward higher genetic gains since its inception almost two decades ago (Crossa et al. 2017). Consequently, its adoption has become integral to modern crop breeding strategies, where genotypic information is used to predict phenotypic traits like crop grain yield. A prediction model learns from existing data (called training set) and then applies that knowledge to predict the traits in new data (called test set). Achieving high prediction accuracies, therefore, relies heavily on training/test set relatedness, and out-of-sample scenarios often result in lower values due to lack thereof (Hickey et al. 2017). One possibility to address the latter is leveraging historic breeding information by aggregating small and intermediate size data (Zhao et al. 2021). However, this remains challenging and largely undocumented for plant breeding domain since most historic data was not archived with interoperability in mind, this is especially true for publicly available data (Papoutsoglou et al. 2023). A shift in perspective toward aggregating data is therefore necessary and opens up opportunities to benefit from myriad of data generated within the domain (Wang et al. 2025; Xu et al. 2022).

Interestingly, while many studies producing such data aim to ensure reproducibility, they often provide only minimal supplementary information, leaving broader aspects of data sharing unaddressed. The term FAIR summarizes guiding principles for scientific data to improve data handling, transparency, and ultimately impact by making the data (1) *findable,* with rich metadata and uniquely indexed in a searchable resource such as domain-specific repositories or general platforms like Google, (2) *accessible,* using a standardized communication protocol like HTTP (Hypertext Transfer Protocol), (3) *interoperable,* through the use of domain-specific syntactic structures and semantic vocabularies (i.e., ontologies), and (4) *reusable*, with clear usage licenses and provenance information (Wilkinson et al. 2016). To support this, the concept of FAIR Digital Object has been proposed to act as a building block (De Smedt et al. 2020) and help shape domain-specific data ecosystem(s) for future research and innovation. But, much needs to be done to cement these blocks, starting with their wider use.

If all publicly available data were truly FAIR Digital Object(s) (FDO), by design, discovering which FDOs to combine for answering specific research questions could, at least in part, be automated. This would be made possible through richly described provenance information and the use of ontologies to detail the data encapsulated within the FDO. In reality, however, these features are only partially available, making it easier to integrate data derived from a single study than from multiple studies. These studies may range from time-limited research activities to long-term breeding programs in public or private domains. The challenge, therefore, is not simply enforcing a top-down approach where “everything is FAIR”, but rather adapting FAIR principles to fit the data management practices commonly observed in the field.

To address this, we define a “Data Cohort” as the collection of various kinds of data generated within a single study. When the data lifecycle adheres to FAIR principles, each kind of data within the cohort could potentially become an FDO. Alternatively, the study might choose to reuse an existing FDO. A Data Cohort, therefore, serves as a structured package of FDOs from or for a study, acting as the primary unit of data availability and exchange in plant breeding research. Expanding on this, we (1) summarize data management steps for creation of FDOs for major kinds of plant breeding data, (2) propose a framework to benefit and identify potential FDO within public data infrastructures to package as Data Cohorts, (3) share experiences in aggregating Data Cohorts for use in genomic predictions, and (4) present an outlook for genomic predictions as part of a data analysis platform to drive future innovation and research in the field.

## Background

### Digital objects as a unit for implementing FAIR

A digital object refers to any type of data shared on a trusted infrastructure—domain-specific repositories for example—with sufficient metadata and a persistent identifier(s) to enable its reuse. FAIR Digital Object (FDO) is an extension of the concept (De Smedt et al. 2020) to satisfy properties of machine (1) interpretability with metadata description(s), (2) interoperability, and (3) actionability. The growing advocacy for FAIRness (Ewert et al. 2023) points to rising awareness within the plant breeding domain and attempts to address (1) siloed nature of studies and (2) sparse and inconsistent availability of domain-specific ontologies (Nédellec et al. 2024).

Ontologies provide a crucial framework for data interoperability by defining domain concepts, i.e., data semantics and their relationships. This is generally done using an annotation model that defines levels of concept abstraction and information aggregation. For example, crop ontology (https://cropontology.org) uses GY_M_kgPlot (variable_id CO_321:0001222) to refer to “Amount (weight) of grains that was harvested” (trait_id CO_321:0000013) after physiological maturity (method_id CO_321:0000236) at a plot level and measured in kilograms (scale_id CO_321:0000806). This hierarchical organization enables efficient data sharing through reusable components while facilitating ontology-driven knowledge discovery through standardized semantic relationships, especially for wide range of research data (Dumschott et al. 2023). Plant variety testing offices, which already define major traits for crop evaluation, represent promising initial sources for developing ontologies that could enable data interoperability, but may need to be adapted for wider use—crop ontology, for example, allows defining new ontologies.

While ontologies may be used to describe the data points, additional attributes on the experiment itself (study metadata) as well as the recorded data point (annotation metadata) may be captured using metadata recommendations like Minimum Information About Plant Phenotyping Experiments (MIAPPE) (Papoutsoglou et al. 2020). MIAPPE provides a standardized list of metadata attributes specifically tailored to describe plant phenotyping experiments (https://github.com/MIAPPE/MIAPPE). To organize this metadata, the attributes can be structured using the Investigation-Study-Assay (ISA) data model (Sansone et al. 2012). The ISA breaks down metadata into three components: (1) the investigation file, detailing study goals and methods; (2) the study file, describing sample metadata, characteristics, and treatments; and (3) the assay file, cataloging qualitative or quantitative data from measurements. These files can be nested, with one investigation file covering multiple study components (e.g., genotypic and phenotypic data from a plant breeding experiment), each linked to its own assay file.

The flexibility of the ISA data model allows multiple file formats for serializations including the ISA-Tab, ISA-JSON (https://isa-specs.readthedocs.io/en/latest), and ISA-XLSX (Weil et al. 2023). ISA-Tab, in particular, has been extensively used for publishing Gene Bank datasets (Gonzalez et al. 2018; Philipp et al. 2019; Schulthess et al. 2022; Svoboda et al. 2024) and multi-environment trial datasets (Gogna et al. 2022), establishing precedence for future data submissions. Over time, data models like ISA and minimal information recommendations such as MIAPPE have been incorporated into FDO constructs to enable automated communication between infrastructures hosting this data (Clarke et al. 2023). One such implementation is Annotated Research Contexts (ARCs), which builds upon the ISA data model, extends it with documentation of computational workflows (Crusoe et al. 2022), and includes data provenance using the Git version control system (Weil et al. 2023).

The increasing adoption of FDOs fuels the development of a FAIR data ecosystem (Hodson et al. 2018) based on: policies that define rules and manage component interactions, data management plans outlining handling of data during a study, persistent identifiers as well as standards that guide FDO creation, and repositories for secure storage. Such an ecosystem would require data infrastructures to be interlinked with ontologies to maintain semantic consistency and shared data meaning across the domain. This will enable users to seamlessly discover and integrate relevant FDOs. For instance, in genomic prediction studies, combining genotype and phenotype data from multiple sources could produce comprehensive datasets, addressing challenges such as limitations in training/test set relatedness (Zhao et al. 2021). More importantly, such an ecosystem could facilitate public–private partnerships for genome-wide predictions as well as incorporation of artificial intelligence methods into plant breeding domain (Lell et al. 2025).

### Data Cohorts define levels for data integration

Data provenance is fundamental to effective data integration, providing crucial context regarding data origin, curation methods, and associated uncertainties. While integrating data within a single Data Cohort is relatively straightforward due to their shared provenance, combining data across multiple cohorts presents significant challenges (Zhao et al. 2021). These challenges stem from variations in experimental designs, data collection protocols, and the potential for incomplete or inconsistent metadata.

**Fig. 1:**
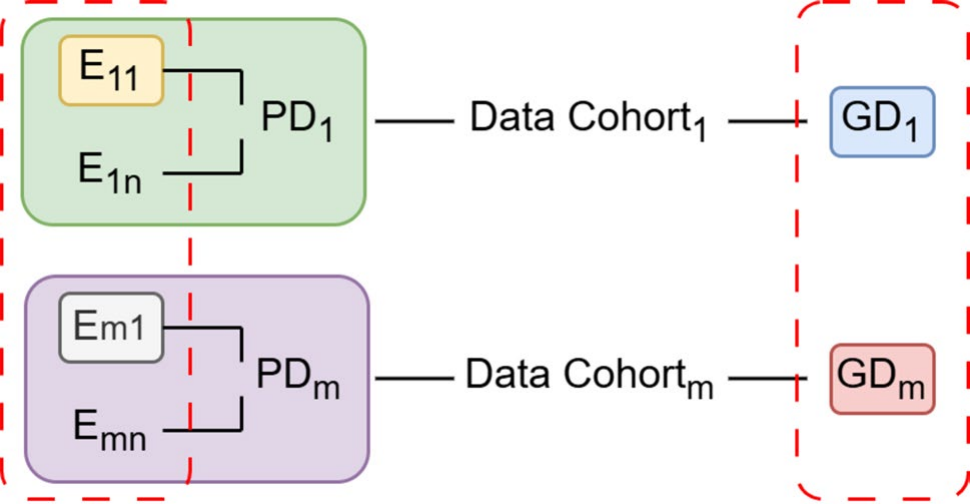
Concept of Data Cohorts: Most plant breeding research combines multi-environment phenotypic data (Green, Purple) with corresponding genotypic data (Blue, Red). This combination of phenotypic and genotypic data is referred to as a “Data Cohort.” To generate Big Data from these cohorts, the red outlines illustrate an integration strategy across environments (E_11_–E_mn_) and genotypic data (GD_1_–GD_m_) (color figure online)

In the context of plant breeding, a common approach to phenotypic data processing involves a stage-wise analysis. Initially, data from each environment (Fig. 1, E_11_–E_mn_) within a Data Cohort (Fig. 1, 1 to m) is independently analyzed to correct for experimental design effects specific to that environment (Fig. 1, yellow box). Subsequently, environment specific effects corrected data within the cohort are aggregated, often using genotype identifiers to merge with genotypic data (Fig. 1, green box). Although automated pipelines for data quality control and preprocessing exist (Chen et al. 2024; Xu et al. 2022), they are not always applicable to the diverse and heterozygous data generation processes typical of plant breeding. Our framework therefore relies on data providers to ensure FAIR compliance, enabling Data Cohorts.

However, to leverage the full potential of historical and diverse datasets for genomic prediction, a more expansive integration strategy is required. This involves integrating data across Data Cohorts, effectively combining environments (E_11_–E_mn_) and genotypic datasets (GD_1_–GD_m_ (Fig. 1, red box)). This cross-cohort integration necessitates rigorous assessment of both data provenance and data veracity (i.e., quality and reliability). While metadata associated with FAIR Digital Object can provide crucial provenance information, a consensus on standardized quality metrics for plant breeding data, particularly for genotypic data, is still evolving. Nevertheless, ongoing efforts to establish such standards (Beier et al. 2022; Hafner et al. 2025) offer a promising path forward.

The successful aggregation of Data Cohorts enables a wide spectrum of downstream analyses. These range from basic investigations of population structure within specific panels to complex studies aimed at dissecting genotype times environment interactions. Therefore, clearly defining the specific use case is paramount. This definition guides the selection of appropriate digital objects and the Data Cohorts that encompass them. This manuscript focuses on utilizing the Data Cohort concept to assemble datasets specifically for developing and refining genomic prediction models. A prerequisite for this is the availability of common, ideally standardized, genotypes in multiple Data Cohorts that enable this connectivity.

### Revisiting data integration for big data in plant breeding

Aggregating Data Cohorts can generate Big Data, defining a collection of large (*Volume*), often very diverse (*Variety*) kinds of data generated at high *Velocity* that require complex analytical methods for processing. The definition may further be refined (De Mauro et al. 2016; Ward 2013), by attributes such as *Veracity*, which refers to the trustworthiness and reliability of both the data and results generated from it. Across diverse fields, the rapid growth in available data has catalyzed a shift toward Big Data methodologies (Ekbia et al. 2015)—a trend vividly reflected in plant breeding today.

In plant breeding, the shift toward Big Data is largely process-oriented, enabling investigations into research questions that were previously constrained by data limitations. For instance, effectively studying genotype times environment (G × E) interactions often requires integrating data from multiple cohorts, as a single study rarely encompasses the full breadth of information needed. In the development of new methods for prediction of genotype performance in novel environments (Washburn et al. 2024), investigations into G × E patterns (Lopez-Cruz et al. 2023) have, for example, benefitted from successful data integrations.

Whether a Data Cohort from a single study qualifies as Big Data remains debatable. A single cohort may include genotypic, phenotypic, and other data types, with the velocity of data generation influenced by the underlying biological or physical processes. For example, while collecting and curating grain-yield data in winter wheat might span an entire growing season (October to August), genotyping data can be produced in just a few days, and climate data may be generated almost in real time. Data produced at higher velocities often contributes significantly to overall data volume, thereby meeting Big Data criteria.

Legacy data can also be transformed to adhere to FAIR data principles (Gogna et al. 2022), and used for building Data Cohorts. However, this process requires significant effort and may yield incomplete Data Cohorts with missing data and/or metadata. In order to address this issue, it is critical to adopt a “FAIR from the beginning” approach (Weil et al. 2023), with a particular emphasis on the establishment of fundamental data models and a comprehensive metadata description within the domain. This should include data pertaining to geno- and phenotypic characteristics, as well as data describing the environmental conditions under which the data was collected. The following sections will explore steps that should be taken with regard to each of the aforementioned data.

### Genotypic data interoperability must account for platform associated ascertainment bias

Genotypic data may be produced using different technologies, each potentially yielding a distinct FAIR Digital Object (FDO). Producing genotypic data involves several steps, from DNA extraction of the organism under research to the actual genotyping, a technique used to identify specific genetic markers or sequences in the genome. Two commonly used technologies are sequencing-by-synthesis (Slatko et al. 2018) and hybridization-based methods, such as SNP arrays. SNP arrays detect allele-specific hybridization, where DNA fragments from (plant) samples hybridize with allele-specific oligonucleotide markers immobilized on a microarray. Fluorescent signals are produced from this hybridization, indicating the allelic state for each marker. These signals are used to identify genotype clusters for respective markers (Wang et al. 2014) and variant data is summarized in, for example, HapMap (Gibbs et al. 2003)-based file formats.

In contrast, sequencing-by-synthesis-based methods, including DArTseq (Elshire et al. 2011), genotyping-by-sequencing (Sansaloni et al. 2011), and whole-genome sequencing (Yano et al. 2016), involve de novo sequencing of DNA fragments, although their sequencing library preparation methods may vary. The raw sequencing data obtained from these methods is typically presented as reads in FASTQ format (Cock et al. 2010) and is commonly processed (Lefouili and Nam 2022) into a variant call format (VCF) format (Danecek et al. 2011). An important distinction from SNP arrays lies in the use of a reference genome to define the variants in VCF-based data. This data may be packaged into an FDO. For this, the provenance information may be stored with header lines for both kinds of data (Beier et al. 2022), while maker-associated metadata may be derived from oligonucleotide information in case of SNP array data (EMBL-EBI 2025) and reference genome in case of VCF-based data.

**Fig. 2:**
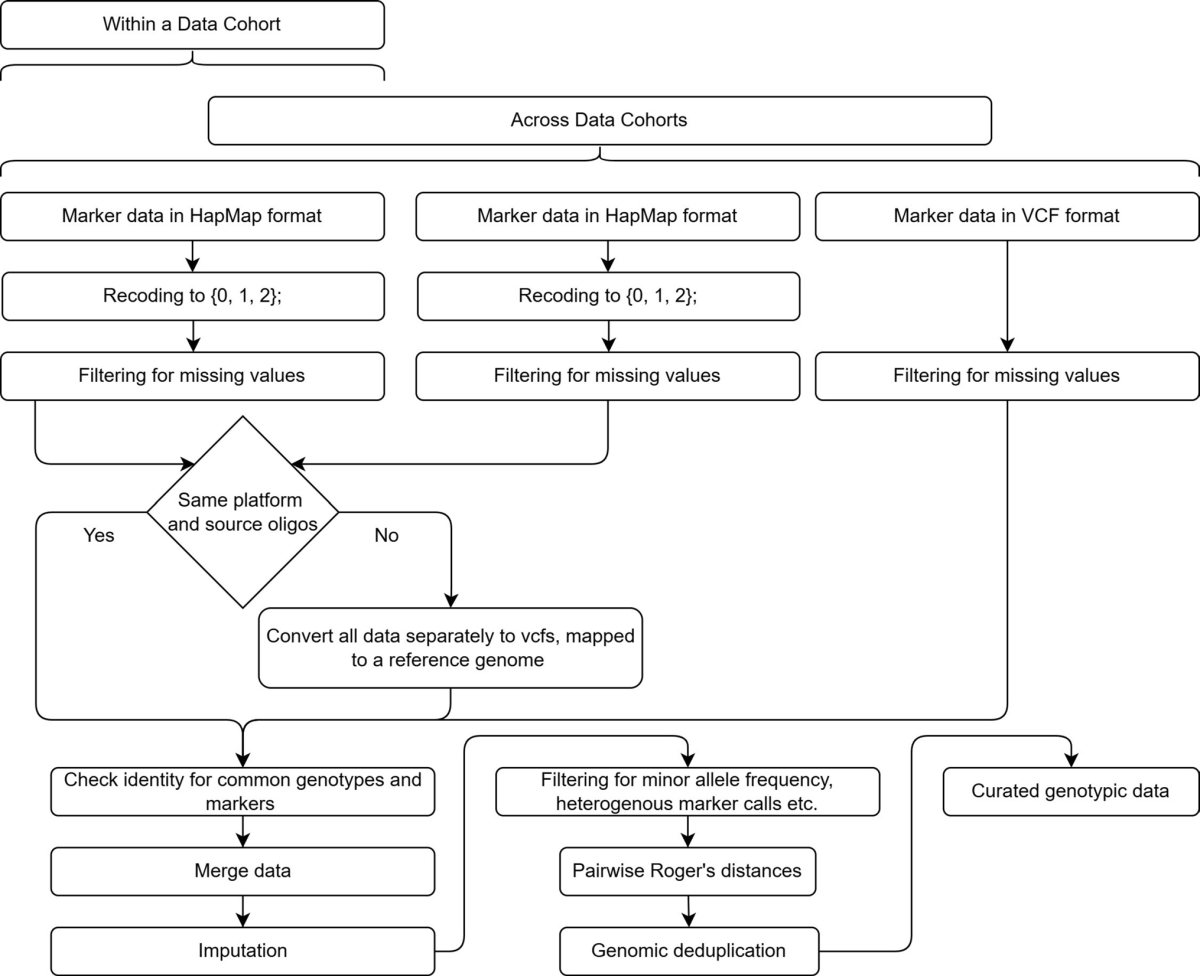
An example decision tree outlining the steps to integrate HapMap and VCF-based data for curating genotypic data

Integration across Data Cohorts, whether based on HapMap or VCF data, may be required to enable broader analyses (Fig. 1). This benefits from shared provenance, overlap of marker variants, and investigated samples across the cohorts. However, additional considerations are crucial during the integration process. For example, when working with VCF-based FDOs, it is essential to ensure that a common reference genome was used for variant calling, e.g., RefSeqV1.1 or RefSeqV2.1 (Zhu et al. 2021) for bread wheat (*Triticum aestivum* L.). Similarly, the integration of HapMap-based FDO generated using different oligonucleotide sets can be challenging due to (1) the proprietary nature of oligonucleotide sequences, (2) the imprecise determination of physical positions for variants captured by oligonucleotide using local alignment (Bethesda 2008), and (3) difficulties in defining variants relative to a consistent reference genome, in species with large genomes and polyploidy (Martin et al. 2022). Moreover, the outcomes of both (2) and (3) are highly dependent on the thresholds applied during alignment and position inference. When integrating multiple HapMap-based FDOs, it is therefore recommended that each of these is individually converted to a VCF-based FDO before integration using overlapping marker positions. This step corrects for any platform-associated bias(es) and, more importantly, allows integrating HapMap-based FDOs with those based on VCF format (Fig. 2).

With integrated data in a VCF format, preprocessing is easily done using vcftools (Danecek et al. 2011) and the genotype calls may directly be converted to integer type representing marker effect values using *plink* (Purcell et al. 2007) for downstream analysis. As a last step, data reduction may be performed to filter out markers with high missing values, low minor allele frequencies, and monomorphic markers. Any missing values for marker variants after preprocessing can be imputed using mean effect values, though complex methods exist (He et al. 2015). Hybrid genotype information may also be derived from parent genotypic data, when needed, at this stage (Zhao et al. 2015).

### Genotypes bridging the individual trials are required for integrated phenotypic analyses

The phenotypic component of a Data Cohort is collected from the field in a given environment, i.e., combination of location and year over the growing season of the crop. Presently, lack of standardization in how traits are recorded is a major restriction to ensuring interoperability across the domain. This is mainly due to differing agronomic practices and logistics involved in data generation. Trait ontologies, for example, crop ontology (https://cropontology.org), address part of this challenge by offering standardized vocabularies and concepts, while constructs like MIAPPE may be adapted for recording of study and annotation metadata from field trials.

Data processing following collection may be organized at multiple levels. Ideally, phenotypic data from each environment needs to be processed individually, which majorly include checks for accurate digitalization of information from the field. Plausibility checks for expected data range, data type as well as patterns of missing information may also be performed. After which, data may be linked to established ontologies and stored following a standardized syntax (syntactic identity) to maintain interoperability across environments. This data may be published online as a FAIR Digital Object, with or without corresponding genotypic data (see previous section).

**Fig. 3:**
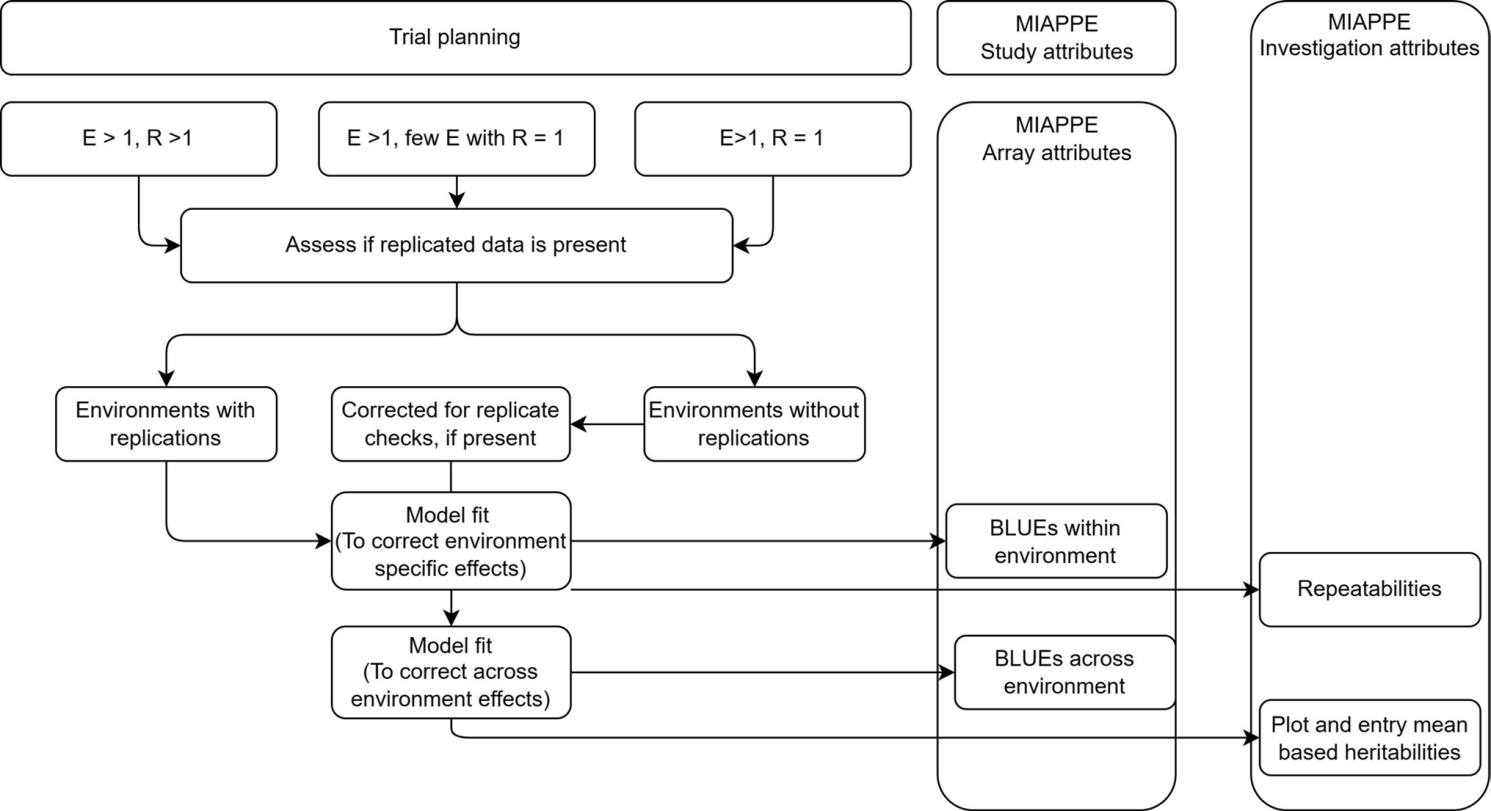
An example decision tree outlining the steps to integrate phenotypic data collected from trials in the field. The abbreviations are as follows: “E” refers to environment, representing a combination of location and year; “R” stands for replication, indicating whether the material in the trial was replicated

The data obtained from each environment, often comprising a single trial, follows a statistical experimental design to allow adjustment for spatial field effects like heterogeneous soil composition, and, management practices, among others. Such corrections are often implemented at the level of trial itself (Fig. 3) using a stage-wise approach (Piepho et al. 2012), although exceptions exist in cases where: (1) trial is unreplicated, and (2) multiple trials are conducted at a given environment (Boeven et al. 2020). The adjusted phenotypic data is referred to as BLUEs. The term “Best Linear Unbiased Estimates” (BLUEs) is used to summarize that the adjusted data represents the most accurate estimation of the true phenotypic value for the genotypes that have been evaluated in the trial. Given that variations in growth environments have the potential to influence BLUEs, it is necessary to distinguish between BLUEs derived at the environment level (stage-one BLUEs) and those derived across environments (stage-two BLUEs). The former essentially correspond to BLUEs for a Data Cohort. Consequently, stage-two BLUEs may also be derived across Data Cohorts, as illustrated in Fig. 1, using following equation:

1 $$y \, = \, \mu \, + \, F\tau \, + {\text{ Ru }} + \, e,$$

where *y* is a vector of stage-one BLUEs ordered as genotypes within respective environments, *µ* represents the overall mean, while *F* and *R* are design matrices for fixed and random effects, respectively. *τ*, *u,* and *e* are vectors of fixed effects, random effects, and residuals, respectively. Generally, genotype effects are considered as fixed, whereas environmental effects are considered as random components in the model, with the assumptions that *u*, *e* ~ (0, Iσ^2^). Additionally, the Data Cohort(s) may also be modeled as a fixed effect in the model (1).

For our approach, we use the phenotypic data corrected for experimental design effects at the trial level as the building block for the phenotypic component of a Data Cohort. While the environments within a single Data Cohort often demonstrate strong connections with check genotypes, this connectivity may not extend across different Data Cohorts. Uniquely identifying genotypes is a key challenge to address when integrating Data Cohorts, especially to avoid string-based identity mismatches. For example, one study might label a genotype as “G7nZ2” while another uses “g7nz2,” resulting in two identifiers that look different to a computer—even though they refer to the same biological entity. One way to resolve genotype identity is by using external databases, such as http://wheatpedigree.net/ for released varieties.

Genotype connectivity across Data Cohorts increases the risk of failing to accurately estimate model parameters, which could potentially lead to issues with model convergence. This connectivity may be artificially introduced using genomic deduplication, if this leads to identical or near-identical genotypes in different Data Cohorts. The idea follows the estimation of genetic distances (Zhao et al. 2021) using integrated genotypic data with proxy identifiers generated for genotypes in the stage-one BLUEs to be integrated. These proxy identifiers are then used to derive genotype effects in model (1).

### Quality metrics ensures that only high-quality phenotypic data enters the integration pipeline

The fit of candidate stage-specific models to the data is typically evaluated using a step-up or step-down approach with the Akaike information criterion (AIC) or the Bayesian information criterion (BIC). Once the optimal model has been identified, an important subsequent step is the phenotypic variance decomposition, which assesses the trait variance architecture (Boeven et al. 2020).

Two important distinctions are vital here, firstly to estimate a parameter for assessing the stability of the phenotype when a given genotype panel is phenotyped in different environments, and secondly to estimate trait heritability. For the former, the term “reliability” has been proposed (Bernardo 2020) as opposed to “repeatability”. For the purposes of this study, the two terms are used interchangeably. Repeatability is calculated akin to heritability and is expressed as the ratio of entry (or genotype) variance to phenotypic variation for a given environment. Phenotypic data with low repeatability values often requires additional investigations before it is used for integrated analysis. This is because low repeatability values indicate major proportion of phenotypic variance being nongenetic in nature. If need be, environments with repeatability values dropping below a defined threshold, for example, 0.3 for grain yield, may be discarded before integrating phenotypic data. This information may be included in the FAIR Digital Object by extending the list of attributes in MIAPPE.

### Genomic repeatabilities for genotypic-phenotypic data interoperability

Similar to repeatability values defined for phenotypic data quality in a given environment, a measure of fit between genotypic and phenotypic data can be derived using SNP-based genomic repeatabilities (Yang et al. 2010). The variance in stage-one BLUEs may therefore be decomposed into additive and additive epistatic components, as follows,

2 $$y \, = \, \mu \, + {\text{ Ru }} + \, e,$$

where *y* is a vector of stage-one BLUEs and the rest of the model terms are similar to those described in (1). In this case, however, the random components are assumed to follow a normal distribution, with *u* ~ (0, $G{\sigma }^{2}$), and *e* ~ (0, $I{\sigma }_{e}^{2}$), where $G$ represents the genomic relationship matrix and *I* is an identity matrix. ${\sigma }^{2}$ is the variance for $G$, representing the additive and additive epistatic effects (${G}_{a}$ and ${G}_{aa}$). These may be derived as follows (Jiang and Reif 2015);

3 $$G_{a} = \frac{{WW^{T} }}{{2\mathop \sum \nolimits_{k = 1}^{p} p_{k} \left( {1 - p_{k} } \right)}},$$

4 $$G_{aa} = G_{a} \# G_{a} ,$$

If $X= ({x}_{ij})$ is a $n \times p$ matrix derived from integrated marker data, where ${x}_{ij}$ represents the number of reference alleles for the $i$^th^ genotype at the $j$^th^ marker, then $W= ({x}_{ij} - 2{p}_{j})$, ${W}^{T}$ denotes the transpose of $W$, and ${p}_{j}$ is the reference allele frequency at the $j$^th^ marker. The symbol “#” denotes a Hadamard product to approximate first degree epistasis interaction effects. Subsequently, narrow sense (R_narrow_) repeatabilities can then be derived using the formula:

5 $$R_{{{\text{narrow}}}} = \frac{{\sigma_{a}^{2} }}{{\sigma_{a}^{2} + \sigma_{e}^{2} }}$$

where ${\sigma }_{a}^{2}$ and ${\sigma }_{e}^{2}$ are variance components derived from model (2) for the additive and error effects respectively. While genomic repeatabilities are reported per environment, the same concept can be extended to stage-two BLUEs, allowing the derivation of a measure of genomic heritability using (2). This information may be included in the FAIR Digital Object by extending the list of attributes in MIAPPE.

### Environmental data allows characterization of crop growth environments

Akin to genotype and phenotype data, the environment in which a trial is conducted can be characterized using climate variables such as precipitation, temperature, and solar radiation (Xu 2016). Other components of the environment, including soil and crop management, may be used to enrich the description of the environmental conditions (de los Campos et al. 2020). Additional information regarding crop growth, like vegetation cover, surface temperature, etc., may be derived from remote sensing geospatial data (e.g., Moderate Resolution Imaging Spectroradiometer data). This data when generated within the course of a study may be packaged into the FAIR Digital Object, given ontologies and metadata attributes, e.g., MIaGIS (Minimum Information about Geospatial Information System) (Thompson et al. 2023), are richly described. For when this is not the case, potential ontology [climate (Eaton et al. 2024), soil (Palma et al. 2020), and crop management (Subirats-Coll et al. 2022)] and minimum attribute list sources [climate [https://gcos.wmo.int/site/global-climate-observing-system-gcos/essential-climate-variables], soil [https://www.fao.org/global-soil-partnership/en/], and crop management (White et al. 2013)] may be adapted for breeding-specific applications.

Notably, climate-related data is often obtained from public resources, such as the Climate Data Center (Kaspar et al. 2019), rather than being recorded on-site. This data is available at various spatial and temporal resolutions and may also be obtained from commercial platforms like ClearAg (DTN 2024) or through environmental sensors deployed in on-site micro weather stations. In the case of the latter, measurement uncertainty—including instrument errors in field measurements and environmental sensor accuracy—must be recorded for meaningful data integration. When multiple data sources are used to describe an environment, the data integration must additionally account for any spatiotemporal patterns and data gaps (Ruane et al. 2015).

Beyond the application of environmental data in the estimation of accurate BLUEs value within or across Data Cohorts (de los Campos et al. 2020), a nuanced understanding of genotype times environment interactions may be achieved by integrating genomic prediction with process-based crop growth modeling tools. However, this would require an additional layer of data to create cross-model-friendly Data Cohort(s), facilitating better collaboration and insights across these domains. This need arises because breeding programs primarily focus on end-point traits like yield and measure only minimal phenological traits due to the large number of genotypes to screen. In contrast, crop models require detailed process-level data for calibration, creating a significant data integration challenge.

Key process data needed includes regular biomass measurements throughout the growing season (with partitioning between plant organs), leaf area index. Phenological observations, like detailed timing of developmental stages, flowering dates, and senescence patterns, are also crucial. Additionally, resource use efficiency metrics, such as radiation interception, water use efficiency, and nitrogen uptake, are needed for comprehensive model calibration. Especially for phenotypic data, the Data Cohort would need to account for data aggregation uncertainty (from combining replicated measurements, scaling between plot and field levels, and integrating across environments) and data quality uncertainty (including missing data, outliers, and potential data entry errors) by adopting clear documentation of data life cycle.

### Clusters minimizing GxE guide selection of environment clusters

Although it is technically feasible to integrate an unlimited number of Data Cohorts to generate Big Data, this integration must fulfill two essential conditions. Firstly, there must be sufficient genotype overlap between each pairwise combination to establish meaningful connectivity of Data Cohorts. Secondly, the integration must be meaningful in exploiting genotype times environment (G × E) interactions, in that it allows clustering similar environments. This second condition serves as a filter, excluding environments that exhibit opposing patterns of G × E interactions relative to the target group of environments. One approach to identify these patterns is by using environmental variables, although more explicit methodologies could be explored. When identified, genomic prediction methods may be extended to account for G × E within clusters of similar environments. This has the potential to enable early selection of promising candidates in breeding programs by incorporating environment information along with genotypic data.

Redefining combinations of Data Cohorts may be exemplified by data generated in Gene Banks. In this instance, the Gene Bank data would represent a single Data Cohort. Gene Banks characterize their accessions in trials that are unreplicated, partially replicated or rarely replicated. The first step would be to derive genotype BLUEs for each environment, with corrections made for the experimental design or genotype replicates, if applicable, followed by the second step of deriving BLUEs for all environments across Gene Banks. In the third step, stage-two BLUEs may be integrated with fixed Gene Bank effects in model (1).

The availability of a curated set of standard reference genotypes included in trials across Gene Banks to establish connectivity and enable comparative analysis is essential for such an integration. Since Gene Banks are responsible for safeguarding genetic diversity, we propose the maintenance and availability of such check cassettes to be taken up by Gene Banks. Furthermore, these cassettes would also enable investigations into G × E interactions and determine groups of Gene Banks for a joint analysis.

### Coordinating research data infrastructures for federated data sharing

**Fig. 4:**
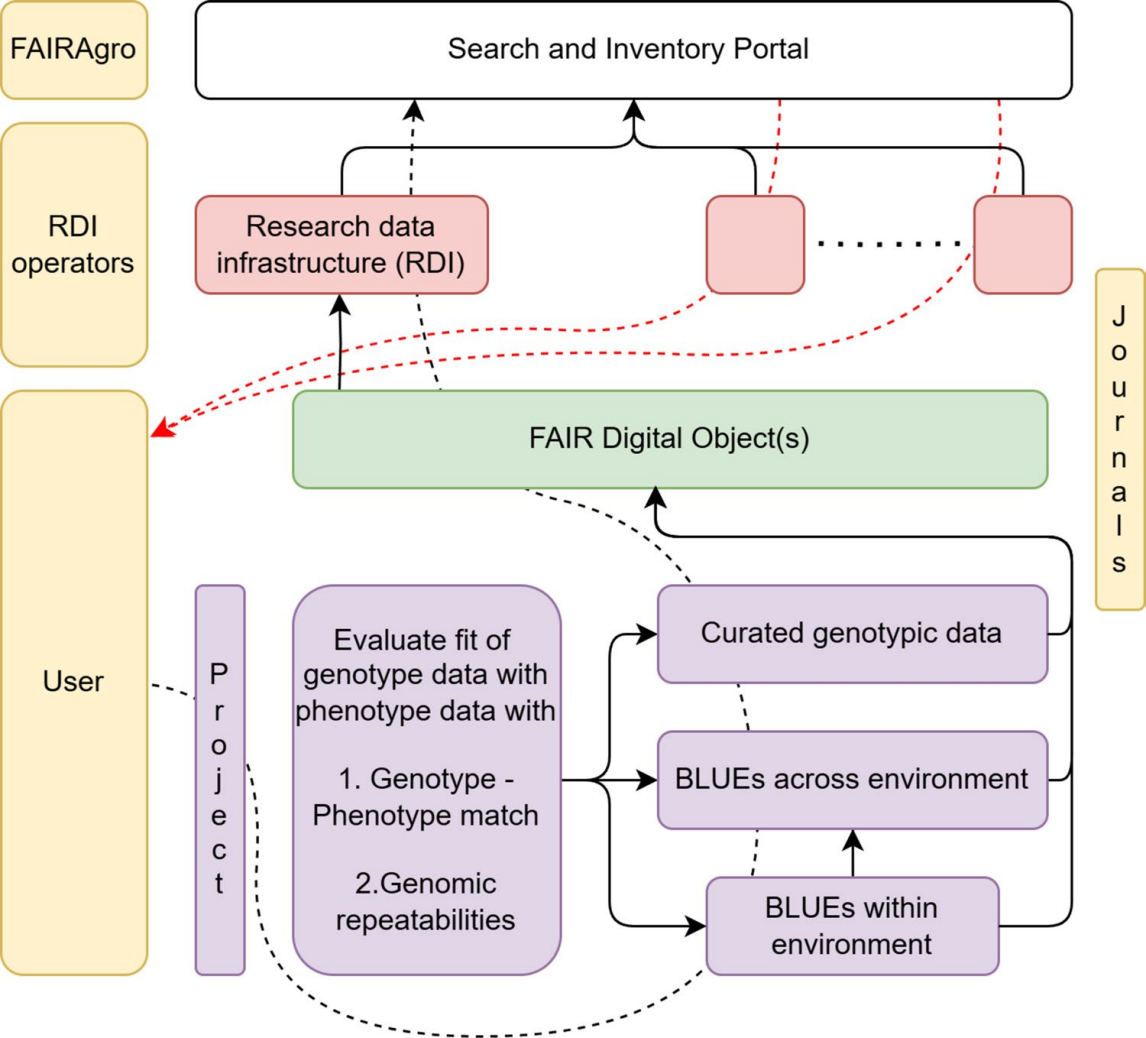
Diagram illustrating how FAIR Digital Objects (FDO) generated within a project (purple boxes) connect to research data infrastructures (RDIs; red boxes) and the Search and Inventory Portal (SIP), which is part of the middleware framework, indexing these FDOs. The yellow boxes represent the agencies responsible for each of these components, viz. users, RDI operators, and larger FAIRAgro consortia. Journals, as represented with a yellow box on the right, are proposed to facilitate the creation of FDOs. The black dotted line represents the typical data flow from users to the SIP, whereas the red lines illustrate an alternative path in which users query the SIP to discover and access indexed FDO

The incorporation of FAIR Digital Object (s) (FDO) into Data Cohorts has, thus far, been described as a task for a user to reflect the current state of the data ecosystem in plant breeding (dotted black line in Fig. 4). Conversely, data federation—which enables seamless communication between different data infrastructures—should allow the design of Data Cohorts in the cloud and their later download for end-use (dotted red line in Fig. 4). This approach effectively circumvents the necessity for manual creation of Data Cohorts, which is essential for expediting the process of knowledge discovery.

Achieving complete data federation requires addressing several challenges, most notably the heterogeneous nature of data infrastructures that are often built using different technology stacks. Furthermore, infrastructures serving the agroecosystem community frequently lack an application programming interface (API) (García Brizuela et al. 2024). To overcome this, the FAIRAgro consortium, which is part of the National Research Data Infrastructure (NFDI) in Germany (Ewert et al. 2023), has proposed the introduction of a middleware concept to streamline (user) access to FDOs (Fig. 4).

It is also crucial to address specific challenges when sharing complex data types, such as matrix data or data with varying temporal resolutions. For these types, it is more practical to associate the data with an external file, rather than cataloging individual data points, for example, within a MIAPPE-compliant format like ISA-TAB (https://isa-specs.readthedocs.io/en/latest/isatab.html; Sect. 2.3.9). This approach, however, means that the data is not immediately machine-interpretable, interoperable, or actionable. To enable this, data infrastructures must expose an API to facilitate communication with other systems. Adopting the MIAPPE mapping to BrAPI (https://github.com/MIAPPE/MIAPPE/blob/master/Mapping/MIAPPE_Checklist_Mapping.tsv) offers a potential solution, supporting the middleware approach discussed earlier and ensuring greater interoperability within the federated infrastructures.

### Journals as custodians for FAIR data ecosystem

The role of journals extends beyond the safekeeping of scientific publications to facilitating data availability for reproducibility of published results. It is becoming increasingly important for publishers to implement clear data sharing policies. Springer Nature, for example, has introduced a four-tier data sharing policy that outlines the requirements for authors wishing to publish in their journals. These requirements reflect the growing scrutiny beginning with (1) data sharing, (2) citing datasets from public repositories, and (3) using data availability statements (Jones et al. 2019). The majority of life science journals in their portfolio adhere to Tier 3, which “strongly encourages” compliance with (1) and (2) while “requiring” (3). Although Tier 4 policies, such as those implemented by *Scientific Data* journal, mandate all three requirements and could support federated data infrastructures, their adoption remains limited. This is primarily due to the increased workload and lack of incentives for authors, along with concerns that these requirements could discourage submissions (Rousi and Laakso 2020).

The transition from “encouraged” to “required” for FAIR submission of data needs to address three key issues: (1) the development of community recommendations, (2) the availability of specialist research infrastructures, and (3) the assessment of submissions. Whereas the first two are already established for at least some data generated within the plant breeding domain, the latter is still in its infancy. One possible solution is to couple FAIR submission with a data descriptor publication, such as in the *Scientific Data* journal. This delegates the responsibility of ensuring data FAIRness to the corresponding data infrastructure, which may automate this process with an assessment tool like FAIR-Checker (Gaignard et al. 2023). These tools, however, check only the “quality” of the infrastructure providing the data, but not the data itself.

In order to comply with increasingly demanding policies for submission of data, it is essential to promote awareness, provide training, and assistance within the community. In Germany, these challenges are addressed through a multi-layered support ecosystem. The FAIRagro consortium (Ewert et al. 2023) provides discipline-specific community workshops and a data steward service center to guide researchers, especially early career researchers, through the data lifecycle (FAIRagro 2025). This is complemented by the foundational bioinformatics resources of the German Network for Bioinformatics Infrastructure (de.NBI 2025) and the high-level recommendations for data management best practices developed by groups like the DINI/nestor Working Group on Research Data (DINI 2025). These national efforts are deeply integrated with broader activities at the European and global levels. For example, the European life sciences infrastructure (ELIXIR 2025) coordinates national activities into pan-European solutions. Together, these infrastructures are key contributors to the overarching vision of the European Open Science Cloud (EOSC 2025), which aims to create a federated web of FAIR data and services for all researchers in Europe. On a discipline-specific international scale, consortia, like AgBioData (Harper et al. 2018), bring together agricultural databases to promote common standards and ontologies.

In the pursuit of building a “FAIR data ecosystem,” (Hodson et al. 2018) funding agencies have also begun requiring data management plans for research projects. These plans outline a proposed timeline and associated steps in the project’s data lifecycle, ideally culminating in the creation of a FAIR Digital Objects (FDOs). For any fruitful use of such an ecosystem, it is essential that the FDOs have a clear reuse license. Reusability is challenging to achieve given the vested interests of data producers against those of potential data users, and successful examples of systems addressing the same are lacking in the plant breeding domain.

### Revising reusability for a federated data ecosystem

Licenses enable a common understanding between data producers and users, regarding potential data use and sharing. Commonly used licenses for data sharing include Creative Commons (https://creativecommons.org/share-your-work/cclicenses/*)* and Open Data Commons (https://opendatacommons.org/licenses/). These, however, are immutable and may lack the necessary flexibility for cases involving proprietary, sensitive, or confidential breeding data, where indiscriminate reuse may infringe on privacy, intellectual property rights, or competitive advantage. This challenge underscores the need for more dynamic, context-aware licensing models—such as tiered access licenses with usage tracking and benefit-sharing mechanisms for data (object) producers and users (called “parties” henceforth).

For data federation to facilitate smooth traffic of FAIR Digital Objects (FDOs), it must address the granular nature of data as well as benefit sharing between parties. We propose that intermediation be taken over by a plant breeding data centric Data Trustee Platform (TP). The TP could also function as a centralized hub for indexing public/private data, as well as a marketplace to identify relevant FDOs for creating corresponding Data Cohorts. Once identified, the TP could additionally facilitate exchange of both information and material between parties via data/material transfer agreements.

As it develops, such a TP must also account for further points arising when data is shared, including security protocols, access control mechanisms, and data governance. Stakeholders from public and private domains should engage in the process to ensure transparent decision-making. This represents a distinct contribution to the plant breeding domain, as we move beyond isolated FAIR datasets to coordinated data ecosystems that can facilitate complex breeding decisions across public and private domains (Lell et al. 2025).

The TP represents a potential business model that extends beyond a purely academic framework and will require further development and validation in the open market. The core components of the TP, however, including API specifications, data cohort assembly tools, and interoperability standards would benefit from being released under an open-source license, to encourage community adoption and collaborative development (Rehm et al. 2021).

To protect proprietary data ownership, the TP might only allow search and discovery, not direct access to the data. To remedy this, the TP would need to be connected to a data analysis platform (AP), designed to operate independently while maintaining on-demand compatibility with the TP. Once legal agreements are in place between parties—a process facilitated by professional data stewards who would manage compliance and assist users—the AP could allow users to access and work with the data indexed in the TP in a defined cloud-based computing environment (https://www.ukbiobank.ac.uk/enable-your-research/apply-for-access). A user can, for instance, upload genotypic data to obtain predictions for a specific phenotype (e.g., grain yield). The AP would then enable the selection of the most relevant Data Cohorts to serve as the training set and perform predictions, which could then be downloaded from the cloud. Importantly, since the AP is independent from the TP, its scope may be extended beyond genomic predictions to embrace continuous improvement for (1) ongoing pipeline optimization, and (2) incorporating feedback from end users to enhance usability and effectiveness.

## Outlook

The shift toward a process-oriented adoption of Big Data in plant breeding requires a fundamental rethinking of data sharing practices. As a first step, transitioning to Data Cohorts with clear licensing could improve data findability, accessibility, and interoperability, thereby easing the innovation bottleneck. This perspective article demonstrates how genomic predictions can leverage such an approach to optimize breeding programs, with winter wheat as an example. Potentially, our framework can be applied to additional crops to broaden the scope of the trusteeship platform. Achieving this requires changes in data policies, discussions on incentives for data producers, and for increased community awareness and training. We aim for this work to serve as a catalyst for these changes and to contribute to a broader cultural shift in data management within the community.
